# A Cross-Sectional Study on Ultrasonographic Measurements of Parotid Glands in Type 2 Diabetes Mellitus

**DOI:** 10.1155/2021/5583412

**Published:** 2021-03-02

**Authors:** Abhishek Gupta, Vijayalakshmi Kondajji Ramachandra, Mubeen Khan, Kumari Sonam Jha, K. S. Vedaraju, Nirmala Alagudu Channaiah

**Affiliations:** ^1^Oral Medicine and Radiology, KIST Medical College and Teaching Hospital, Lalitpur 44705, Nepal; ^2^Oral Medicine and Radiology, Government Dental College and Research Institute, Bangalore 560001, India; ^3^Department of Radiodiagnosis, Bangalore Medical College and Research Institute, Bangalore 560001, India; ^4^Department of Medicine, Bangalore Medical College and Research Institute, Bangalore 560001, India

## Abstract

**Background:**

Diabetes mellitus is a metabolic disease which is seen increasing globally and is diagnosed and monitored on basis of invasive blood investigations. Salivary glands are affected in diabetes mellitus. The objective of this study was to assess ultrasonographic measurements of parotid glands and correlate with the glycosylated hemoglobin levels in type 2 diabetic mellitus and duration of type 2 diabetic mellitus and treatment regimens.

**Materials and Methods:**

This study was conducted on 50 subjects of type 2 diabetes mellitus and on 50 healthy controls. After HbA_1C_ analysis of selected individuals, 100 individuals were grouped into group I (above 5.7) and group II (below 5.7). Ultrasonographic measurements (length (*L*), transverse dimension (TD), depth lateral to the mandible (DLM), and depth dorsal to the mandible (DDM)) of bilateral parotid glands were calculated. Statistical analysis was done using the chi-square test of significance and Spearman correlation coefficients.

**Results:**

On correlation with measurement of right (L, DLM, DDM) and left (TD, DLM, DDM) of parotid glands with duration of type 2 diabetes mellitus, we found a moderate positive relationship, whereas as for right (TD) and left (*L*), we found a low-positive relationship. Similarly, for right (L, TD, DLM, DDM) and left (TD, DDM) parotid glands with HbA_1C_, we found a low-positive relationship, whereas for left parotid gland (L, DLM) with HbA_1C_, we found a moderate positive relationship. The mean DLM of right and left parotids in the insulin group was found to be slightly more than that in the combined group which was statistically insignificant.

**Conclusion:**

Ultrasonographic measurements of parotid glands were found to be higher in study subjects as compared to control subjects, and they increased with increased HbA_1C_ levels; also, there was no difference in treatment regimen. Ultrasonography could be a prospective diagnostic test for detection and monitoring of diabetes mellitus, and still further studies are required for this.

## 1. Introduction

Diabetes mellitus (DM) is a metabolic disease characterized by hyperglycemia and dysregulation of carbohydrate, protein, and lipid metabolism [1]. Globally, the prevalence of diabetes has doubled from 4.7% (1980) to 8.5% (2014) in the adult population [2]. Diabetes is continuously increasing in India with more than 62 million diabetic individuals currently diagnosed with the disease and a maximum increase to 79.4 million individuals by 2030 [3]. DM is categoried into four types, amongst which type 2 diabetes makes up about 85–90% of all cases.

This affects almost all tissues in the body, and salivary glands are not spared. Diabetes mellitus can have variable involvement in parotid glands affecting the oral health. Prolong standing diabetes mellitus (DM) can lead to glandular hypofunction, xerostomia, and noninflammatory, nonneoplastic enlargement of PGs due to acinar atrophy and fatty infiltration [4]. Salivary glands and saliva have shown changes that can be used for diagnosis and prognosis of various diseases and conditions of oral cavity such as periodontitis which further can be correlated with metabolic conditions such as diabetes [5–7]. Various salivary markers have been found to aid in diagnosis of various systemic and oral diseases including diabetes mellitus [5–7].

Salivary gland involvement in diabetes mellitus can be assessed by several imaging modalities including sialography, computed tomography (CT), magnetic resonance image (MRI), and ultrasound (US). Diagnostic ultrasound (US) is established as first choice of imaging modality for parotid glands [4, 8]. It is a noninvasive procedure using very high frequency (7.5 MHz) pulsed ultrasound beam rather than ionizing radiation to produce high resolution images of salivary glands [8]. On ultrasound, the echotexture of salivary glands varies depending on dominant histological changes (acinar atrophy and fatty infiltration) and overall enlargement of salivary glands can also be diagnosed [9].

Currently, a glycosylated hemoglobin (HbA1c) test has been recommended as a faster, easier diagnostic test for diabetes mellitus [10] but with the disadvantage of being an invasive procedure. Concurrently, some researchers have suggested that salivary composition and function have a potential to contribute to the clinical diagnosis and staging of diabetes mellitus, and it has been proposed that asymptomatic parotid gland enlargement warrants a search for diabetes mellitus [4, 9, 11, 12]. Although many studies have reported the enlargement of parotid glands on ultrasound in patients with diabetes mellitus [4, 11, 12], not many clinical studies, which have evaluated the relationship of hyperglycemic status of type 2 diabetes mellitus and the salivary gland changes, have been conducted in India. Hence, there is lack of studies and consensus on the possible association with salivary gland findings in diabetes mellitus. Realizing this paucity, this study was designed to evaluate changes in ultrasonographic measurements of parotid glands in patients with type 2 diabetic mellitus and their association with the hyperglycemic status. The null hypothesis to be tested was that there is no change in size of parotid glands in patients with diabetes mellitus with duration and hyperglycemic control. The findings of these studies will possibly help us in further considering ultrasonography of parotid glands in diagnosis of type 2 diabetes mellitus.

## 2. Materials

### 2.1. Materials

The materials required are as follows:

  Armamentarium for general physical examination:
(i) Weighing scale
(ii) Height measuring scale
  Armamentarium for clinical examination of orofacial region:
(i) Dental chair with adequate artificial illumination facility
(ii) Disposable mouth masks
(iii) A pair of disposable gloves
(iv) Plane mouth mirrors
(v) Straight probes
(vi) Sterilized stainless steel kidney tray
(vii) Tweezers
  Armamentarium for collection and transport of blood samples:
(i) Sterile alcohol preps or Betadine (povidone-iodine) swab
(ii) Disposable gloves (nonlatex)
(iii) Tourniquet
(iv) Disposable, 21-gauge needle
(v) Disposable, needle holder
(vi) Vacutainer k3 EDTA tubes
(vii) Sterilized gauze pads
(viii) Sterile Band-Aid
(ix) Eppendorf (for storage of serum samples)
(x) Freezer (for storage of serum samples at –200°C)
(xi) Blood transporter box (ice box)
  Armamentarium for HbA1c analysis:
(i) *D*-10 hemoglobin analyzer
  Armamentarium for ultrasonography imaging of parotid glands:
(i) Philips IU22, Siemens Avanto, Ultrasound Machine
(ii) Transducer with high frequency (5–13 mhz).
(iii) A pair of disposable gloves
(iv) Ultrasound gel
(v) Disposable tissue papers

### 2.2. Sample Size Estimation

A pilot study was conducted among 10 subjects to calculate the sample size and to check the feasibility of the study.

Based on the prevalence of at least one parotid gland enlargement in diabetes mellitus type 2 patients, the sample size was calculated.

#### 2.2.1. Formula

(1) $$\begin{array}{c} N=\frac{Z\alpha /2^{2}\times P\left(1-P\right)\times D}{E^{2}}, \end{array}$$

where *P* = prevalence of at least one parotid gland enlargement, i.e., 80%, statistical power = 80%, *Zα* = 1.96 at 95% confidence interval, *E* = margin of error-10%, and *D* = design effect-1.

(2) $$\begin{array}{c} N=\frac{{\left(1.96\right)}^{2}\times 0.8\left(1-0.2\right)\times 1}{{\left(0.08\right)}^{2}}. \end{array}$$

The calculated sample size was 100. Hence, a minimum of fifty type 2 diabetes mellitus and fifty healthy participants were needed to conduct the study.

### 2.3. Source of Data

This study was conducted on 50 subjects of type 2 diabetes mellitus and on 50 healthy age- and sex-matched controls who were selected based on the selection criteria during the period of December 2015 to July 2017 visiting Department of Oral Medicine and Radiology, Government Dental College and Research Institute, Bangalore and Diabetic Centre, Bangalore Medical College and Research Institute, Bangalore. The study was conducted in full accordance with ethical principles and was reviewed and approved by an ethical board of the institution.

  Inclusion criteria for study subjects
(i) Patients in age group between 18 and 70 years
(ii) Patients having body weight in range of 20% of ideal body weight (IBW), according Broca's formula (ideal body weight in kilograms = height in centimeters-100)
(iii) Patients diagnosed with type 2 diabetes mellitus on the basis of clinical and laboratory findings (FBS ≥ 126 or HbA_1c_ >6.5%) according to ADA (2015) for a minimum of 2 years
  Inclusion criteria for control subjects
  Patients visiting the Department of Oral Medicine and Radiology, Government Dental College and Research Institute, fulfilling the following criteria:
(i) Age- and sex-matched subjects with that of the study group
(ii) Patients having body weight in range of 20% of ideal body weight (IBW), according Broca's formula (ideal body weight in kilograms = height in centimeters-100)
(iii) Subjects without history of diabetes mellitus
(iv) Subjects with HbA_1c_ less than 5.7
(v) Subjects without any salivary gland disease
  Exclusion criteria for study and control subjects
(i) Patients with history of parotid gland enlargements such as recurrent parotid gland infections, cyst, tumor, sialadenitis, amyloidosis, sarcoidosis, tuberculosis, Wegener's granulomatosis, and Sjogren's syndrome
(ii) Patients with HIV
(iii) Pregnant and lactating women
(iv) Patients with known cardiovascular or other systemic diseases such as hypothyroidism
(vi) Patients with a history of chemotherapy or radiation therapy for head and neck region
(vii) Patients with habit of tobacco chewing, smoking, chronic alcoholism (>2 units), anorexia nervosa, or bulimia
(viii) Patients under steroids, immunosuppressive drugs or cytotoxic drugs, and drugs influencing salivary glands secretions

## 3. Method of Collection of Data

50 patients of type 2 DM and 50 controls, who had body weight in 20% in the range of ideal body weight (IBW) according to Broca's formula (IBW in kilograms = height in centimeters-100) along with the abovementioned inclusion and exclusion criteria, were included in this study. All the selected individuals were informed about the details of the study in their known local language, and a written informed consent was obtained. A detailed case history, thorough clinical and orofacial examination, was then carried out and documented on a specially designed case history proforma for all the selected individuals.

### 3.1. Method

All the 100 selected patients were subjected to blood sample collection procedure.

#### 3.1.1. Procedure for Collection of Blood Sample

All the 100 selected patients were made to sit on the chair in a relaxed position. The antecubital fossa of the arm was palpated to locate the median cubital vein. Under proper aseptic conditions and a well-illuminated light source, a sterile single use 5 ml syringe and 26 gauge needles were used to draw 2 ml of venous blood sample from the median cubital vein. The collected blood sample was stored in an anticoagulant (ethylene diamine tetra acetic acid) to prevent it from clotting and transferred to the haematology laboratory. The collected blood sample was analyzed for HbA_1C_.

#### 3.1.2. Procedure for HbA_1c_ Analysis

The collected blood sample was transported in a blood transporter box (Ice Box) to Laboratory of Pradhan Mantri Swasthya Suraksha Yojana (PMSSY) in Victoria Hospital Campus, Bangalore. HbA_1C_ analysis was carried out for the collected sample using HPLC HbA_1C_ analyzer on the same day of withdrawal and transport.

After HbA_1c_ evaluation of the selected Individuals, they were divided into the following:

  Group I: 50 known cases of type 2 diabetes mellitus for 2 years and HbA_1C_ level above 5.7
  Group II: 50 nondiabetic patients with HbA_1C_ level below 5.7

#### 3.1.3. Procedure for Ultrasonography of Parotid Glands

All the 100 selected patients (50 type 2 diabetic and 50 nondiabetic) were subjected to ultrasonography of parotid glands in The Department of Radiodiagnosis, BMCRI, Bangalore, by an expert ultrasonographer who was not aware of the diabetic status of the patients. The patients were made to lie down on a mobile table that could be easily positioned so that the patient's neck is in level with the US monitor and within the operators scanning range. The posterior portion of head of patients would be facing towards the table and patients looking up. The patient was explained about the procedure. An ultrasonographic gel as per the requirement was slightly squeezed from a bottle and was applied over the skin surface of parotid glands. A transducer (linear, 7.5–10 mhz probe with a small “footprint”) was used to spread the ultrasonographic gel over the region. The transducer was slowly moved over the parotid region beginning from anterior most region, i.e., preauricular region, and moving towards the postauricular region parallel to the posterior border of mandibular ramus, followed by moving the transducer above till upper border of the gland, from zygomatic arch and external acoustic meatus until the lower border of the gland (at the angle of the mandible).

**Figure 1:**
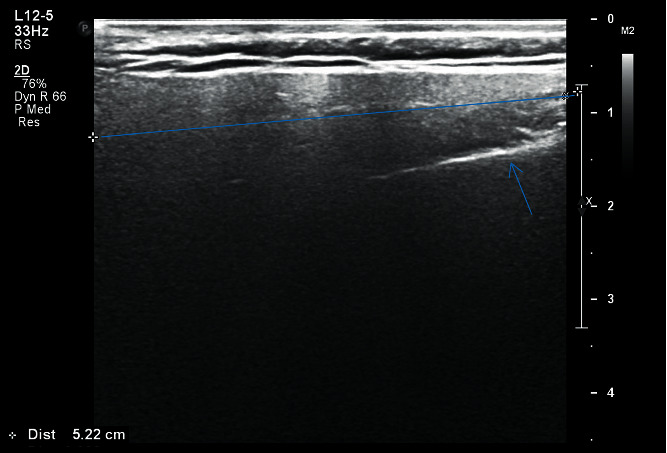
Measurement of length of right parotid gland of patient with HbA1c 9.7.

**Figure 2:**
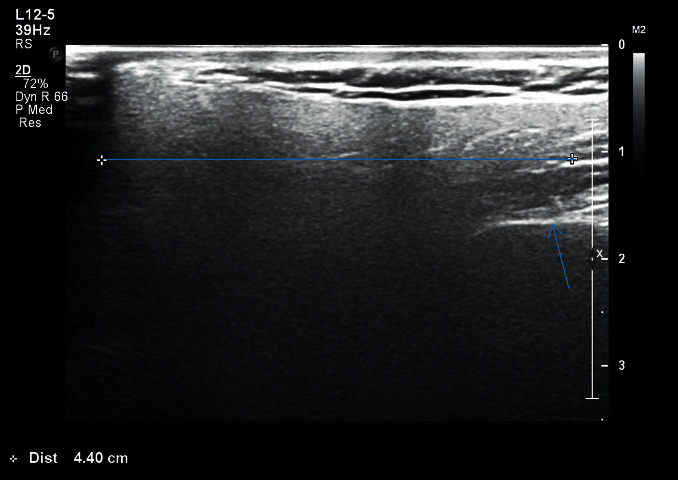
Right parotid gland measurement of transverse length of patient with HbA1c 9.7.

**Figure 3:**
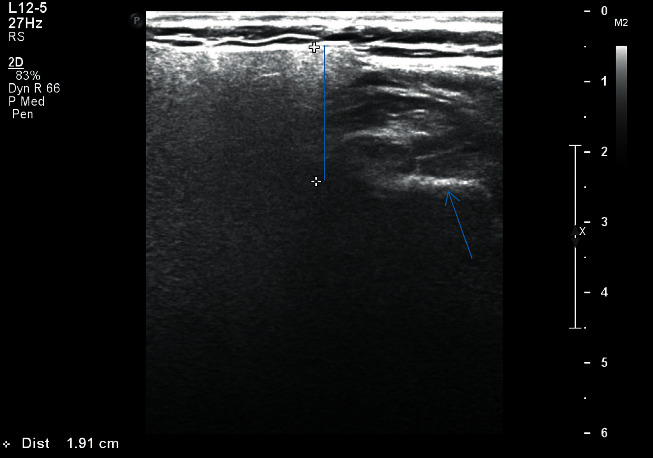
Right parotid gland measurement of depth lateral to mandible of patient with HbA1c 9.7.

**Figure 4:**
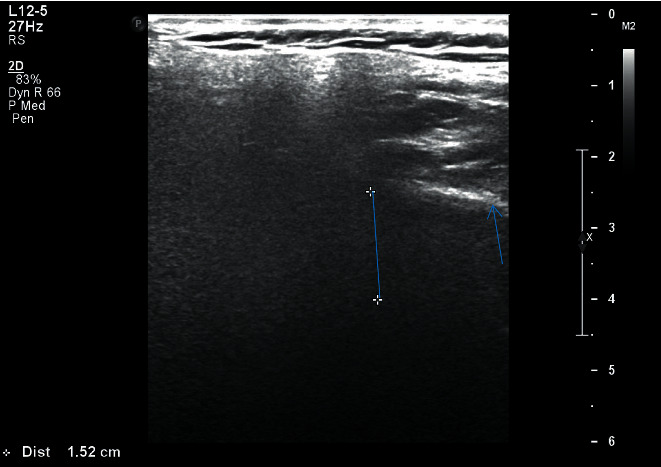
Right parotid gland with measurement of depth distal to mandible of patient with HbA1c 9.7.

The image thus obtained was being monitored simultaneously on the screen of the ultrasonographic machine and was recorded in centimeter scale on a specially designed proforma for both right and left sides separately:

1. Length (*L*): distance between upper border of the gland, from the zygomatic arch and external acoustic meatus until the lower border of the gland till angle of the mandible (Figure 1)
2. Transverse dimension (TD): distance from the masseter muscle and posterior edge of mandibular ramus anteriorly and extended into the tip of mastoid process posteriorly (Figure 2)
3. Depth lateral to the mandible (DLM): depth of parotid gland parenchyma, distance from the subcutaneous region to a depth lateral to the mandible (Figure 3)
4. Depth dorsal to the mandible (DDM): depth of parotid gland parenchyma, distance from the subcutaneous region to a depth dorsal to the mandible extending till retromandibular vein (Figure 4)

Other abnormalities, if any found, during the ultrasonographic examination were also recorded. The entire procedure was done for both the parotid glands. After the completion of the procedure, tissue papers were used to remove the ultrasonographic gels over the skin.

#### 3.1.4. Method of Statistical Analysis

The following methods of statistical analysis have been used in this study. Data were entered in Microsoft excel and analyzed using SPSS (Statistical Package for Social Science, Ver.10.0.5) package. The results were averaged (mean ± standard deviation) for continuous data and number, and percentage for dichotomous data is presented in tables and figures.

1. Associations were compared using the chi-square test of significance.
2. Spearman correlation coefficients were calculated to determine whether there was any correlation between ultrasonographic measurements and diabetes.

The Pearson product-moment correlation coefficient is calculated to measure the strength of the linear relationship between two variables.

In all the above tests, a “*p*” value of less than 0.05 was accepted as indicating statistical significance.

## 4. Results and Discussion

Diabetes is an important public health problem, and the prevalence of diabetes has been steadily increasing over the past few decades [2]. Type 2 DM accounts for almost 80–95% of diabetes mellitus. Type 2 diabetes mellitus affects almost all tissues in the body and is associated with significant complications of multiple organ systems. Diabetes mellitus can have variable involvement of PGs (non-neoplastic enlargement of PGs), and it can contribute to the clinical diagnosis and staging of DM [4, 9, 11, 12].

Also, salivary gland changes and changes in saliva lead to periodontitis which itself have been a proven diagnostic sign for diabetes mellitus [4–7, 12]. Salivary markers already play a vital role in early detection of various oral and systemic diseases [5–7]. Thus, salivary gland changes would independently aid in diagnosis of diabetes mellitus [4–7, 12].

In this study, Broca's formula was used to exclude the patients with obesity, as a significant PG enlargement in patients with adiposity or eating disorders has been found in comparison to normal-weight, healthy participants [9]. The same criteria have been followed in study conducted by Fattah et al. [4] and Dost and Kaiser [11].

### 4.1. Genderwise Distribution of Study Subjects and Control Subjects according to Body Weight and Height (Taken for Selection on Basis of Broca's Formula)

The mean body weight in males (54%) and females (46%) of the study group was 71.72 ± 6.42 kg and 60 ± 6.35 kg, respectively, and that in the control group was found to be 69.42 ± 6.97 kg and 58.30 ± 6.58 kg, respectively.

The mean height in males and females of the study group was found to be 166.43 ± 6.43 cm and 149.87 ± 8.89 cm and that in the control group was found to be 161.69 ± 11.49 cm and 145.39 ± 14.35 cm, respectively. The difference in height and weight in study and control groups was not statically significant with a *p* value of 0.52 and 0.062, respectively.

### 4.2. Age (Groups), Gender, and Type 2 DM

Control subjects and study subjects were in the age range of 33 to 68 years (51.98 ± 9.82 years) and 23 to 70 years (52.86 ± 9.96 years), respectively. In study subjects, 2% (21–30 yrs), 10% (31–40 yrs), 14% (41–50 yrs), and maximum, i.e., 62% were in age group of >50 yrs which was in accordance with study conducted by Mohan et al. [13]. Our study has shown a slightly lower mean age as compared to earlier studies conducted by Ramachandran et al. [14] which can be explained as type 2 DM is now being shown to be occurring at a relatively younger age [3, 10, 15]. The reason behind this is being the steady migration of people from rural to urban areas, the economic boom, and corresponding change in lifestyle [16]. There were equal numbers of males 27 (54%) and females 23 (46%) in study and control groups as they were sex-matched. Therefore, there was no statically significant difference in the gender distribution with the *p* value of 1. Among cases, there was statically significant increase in males, which was in accordance with most of the studies [10, 13, 15] but not in accordance with study by A. Ramachandran et al. [14] which showed no gender difference. The high prevalence in males could be attributed to smoking (elevated for about 10 years after smoking cessation), abdominal obesity, and urbanization [16].

### 4.3. Distribution of Study Subjects according to Duration of Type 2 Diabetes Mellitus and Treatment Regimen

The mean duration of type 2 diabetes mellitus was found to be 8.04 ± 5.1 years ranging from 2 years to 30 years. The mean duration in age group of 21–30 yrs was found to be 2 years with 1 male and 0 female. The mean duration in age group of 31–40 was 3.6 ± 1.4 yrs (*N* = 5) (male (*N* = 3) with 2.67 yrs and female (2) with 5 yrs ± 0). The mean duration in age group of 41–50 yrs was 6.92 ± 3.3 yrs (*N* = 13) (male (*N* = 6) with 5.5 ± 1.3 yrs and female (7) with 8.14 ± 4.2 yrs). The mean duration in age group of >50 yrs was 9.41 ± 6.4 yrs (*N* = 31) (male (*N* = 17) with 10.18 ± 7.2 yrs and female (*N* = 14) with 8.5 ± 5.4 yrs). Among 50 cases, 5 (10%) subjects were under combined treatment of insulin and oral hypoglycemics among which 3 (6%) were males and 2 (4%) were females.

### 4.4. Age, Gender, and HbA_1C_ Level

4% (1 male and 1 female) of the type 2 diabetic mellitus patients had controlled diabetes, and they were above 50 yrs of age. Remaining 96% (26 males and 22 females) of the type 2 diabetic patients had uncontrolled diabetes, i.e., HbA_1C_ >6.5. HbA_1C_ levels of the males in 31 to 50 yrs age group were significantly higher than those of the female group. These observations were in accordance with other studies [17–21]. Women in postmenopausal age groups (>50 yrs) in our study had a steeper slope than men (mean HbA_1C_ of 9.54 in females and 8.62 in males), which is in accordance with study done by Ma et al. [17]. The reasons for the above observations are most likely due to factors such as blood pressure and blood lipids of males in this age group (31–50 yrs) have worse control conditions, and women may be easily affected by physiological cycle. Furthermore, in different gender groups, HbA_1C_ levels gradually rose with increasing age. Also, it is possible that this finding is related to lower hemoglobin levels in menstruating women with more rapid erythrocyte turnover, as suggested by study done by Ma et al. [17].

### 4.5. Age, Duration of Type 2 Diabetes Mellitus, and Glycemic Control (HbA_1C_ Level)

Mean HbA_1C_ levels were 8.8, 9.02, 10.02, and 8.793 in the age groups 21–30, 31–40, 41–50, and 50+ years, respectively. This implies that good glycemic control increased with age. Also, longer duration of diabetes (≥5 years) and treatment with oral agents or insulin were inversely related to good glycemic control. This observation is in accordance with study done by Gilliland et al. [22] and Al-Lawati et al. [23]. This could be due to either “cohort effect,” where various age groups represent different birth cohorts and same differences would continue as cohort's age. Alternatively, it could be due to “developmental effect,” where cohorts mature and their glycemic control improves as they age. The former concept is favored as HbA_1C_ deteriorates with time. However, follow-up studies are more likely to clarify this effect than cross-sectional studies like the current one.

### 4.6. Ultrasonographic Measurements and Age and Gender (Figure 5)

In our study, in both the groups (control and study), there was no significant differences in dimensions between left and right PGs. This observation was in accordance with study connected by Fattah and Zainab [4], Dost and Kaiser [11], and Al-Ubaidy et al. [12]. Slight increase in the overall dimensions of the both right and left PGs was observed in age groups >50 yrs as compared to other age groups which was not statically significant. The mean measurements of right and left PGs in the control group were found to be slightly increased in males as compared to females except for mean transverse dimension of right-side PGs which was slightly more in females as compared to males (not statically significant). Above observations were in accordance with the study conducted by Fattah and Zainab [4], Dost and Kaiser [11], and Topaketal et al. [24] The reason for the increase in dimensions of PGs along with age could be attributed to fatty infiltration and increase in fibrosis of PGs along with age.

In the study group, the mean measurements of length, transverse dimension, DDM in right PGs, and DDM in left PGs were slightly more in females as compared to males which was again not statically significant which was in accordance with the study done by Riyaz et al. [8]. This could be explained by the finding that the diabetic status of the females (average HbA_1C_ level 9.55) was more uncontrolled as compared to males (average HbA_1C_ level 8.78).

Overall regarding the age group, the mean US measurements among study subject were always higher than the control group in all the age groups, and this was in accordance with the study conducted by Riyaz [8]. Overall, the mean dimensions of PGs in both right and left sides were found to be significantly higher in study subjects as compared to control subjects. Above observations were in accordance with the other studies [7, 12, 25–28]. In contrast to our study, Topak et al. [24] in their study failed to find any significant difference in measurements of PGs between the two groups (cases and controls). The principal cause of the increase in glandular size could be the adipose stroma infiltration [16, 26, 29] (both in the acinar as well as ductal cells of PGs) as there was no evidence of inflammatory processes that could justify the PG hypertrophy. In various other studies [18, 26], it has also been suggested that the diabetes induces neuropathic changes in the salivary gland parenchyma and presence of autoimmune lymphocytic gland infiltrate that is similar to the one occurring in the pancreas of these patients, causing the enlargement of the PGs.

No other peculiar changes were seen like calcifications, vascularity, and dilated ducts; thus, sialadenosis on ultrasonography appeared as a mere enlargement of the gland as observed in a study done by Sridhar and Gnanasundaram [30].

Correlation between ultrasonographic measurements of parotid glands with a duration of type 2 diabetes mellitus in study subjects was not mentioned in other studies so could not be compared (Table 2).

### 4.7. Ultrasonographic Measurements and Hyperglycemic Control (HbA_1C_ Levels)

Increased HbA_1C_ levels were associated with increased ultrasonographic measurements which were in accordance to a study conducted by Rangdhol et al. [31] (Table 3). The reason for this observation could be the fact that HbA_1C_ level is directly proportional to the hyperglycemic status of diabetes and chronic hyperglycemia leading to the structural changes in salivary gland including acinar hypertrophy and adipose infiltration [8, 16, 26].

### 4.8. Ultrasonographic Measurements and Treatment Regimen

Among 50 cases of diabetes mellitus, 5 (10%) were under insulin plus oral hypoglycemic drugs and 45 (90%) were under only oral hypoglycemic (Table 4). The difference between mean length of right and left PGs in the insulin group and in the combined group was found to be statically insignificant. Thus, our study concluded that there was no overall significant difference in ultrasonographic measurements due to differences in treatment regimen. Similar observation was seen in study conducted by Al-Mashhadane [32].

## 5. Limitations

The limitations of our study were that it was done under a small group of population and most of them had uncontrolled type 2 diabetes mellitus.

## 6. Conclusions

It was concluded that there is no significant difference in ultrasonographic measurements due to differences in treatment regimen, there was moderate correlation found between ultrasonographic measurements of parotid glands with duration of type 2 diabetes mellitus, and increased HbA_1C_ levels were associated with increased ultrasonographic measurements of parotid glands.

Our study demonstrated a positive relation between the parotid gland enlargement in patients with type 2 diabetes mellitus; however, further studies on larger cohort samples are required for assessing the sensitivity and specificity of this technique in determining the diagnostic significance.

## Figures and Tables

**Figure 5:**
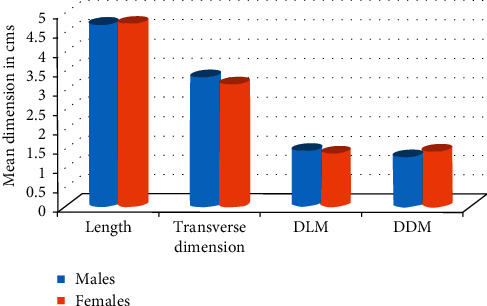
Graph showing comparison of ultrasonographic measurements of parotid glands in males and females in study subjects. Ultrasonographic measurements in study and control subjects (Table 1 and Figure 6).

**Figure 6:**
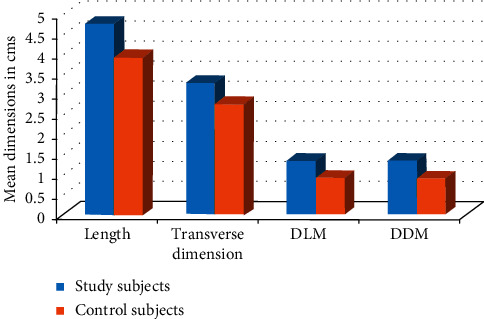
Graph showing comparison of ultrasonographic measurements of parotid glands in study subjects to control subjects.

**Table 1:** Comparison between ultrasonographic measurements in study subjects (type 2 diabetes mellitus) and control subjects.

Type 2 diabetes mellitus	Length (in cms)				Transverse dimension (in cms)				DLM (in cms)				DDM (in cms)			
	R		L		R		L		R		L		R		L	
	N	Y	N	Y	N	Y	N	Y	N	Y	N	Y	N	Y	N	Y
N	50	50	50	50	50	50	50	50	50	50	50	50	50	50	50	50
Mean	3.9	4.74	3.9	4.71	2.73	3.22	2.78	3.33	0.93	1.38	0.9	1.47	0.90	1.36	0.88	1.33
SD	0.66	0.81	0.77	0.89	0.98	1.17	1.10	1.31	0.50	0.56	0.41	1.17	0.58	0.58	0.56	0.55
*p* value	0.001^*∗∗*^		0.001^*∗∗*^		0.04^*∗*^		0.06		0.001^*∗∗*^		0.09		0.001^*∗∗*^		0.0001^*∗∗*^	

^*∗∗*^Highly significant *p* value; ^*∗*^significant *p* value.

**Table 2:** Correlation between ultrasonographic measurements with duration of type 2 diabetes mellitus in study subjects.

	Length (in cms)		Transverse dimension (in cms)		DDM (in cms)		DLM (in cms)	
	R	L	R	L	R	L	R	L
Duration of T2DM	0.22	0.17	0.18	0.21	0.25	0.29	0.24	0.31

0–0.2, low correlation; 0.2–0.4, moderate correlation; 04–0.7, high correlation; >0.7, strong correlation.

**Table 3:** Correlation between ultrasonographic measurements of parotid glands of study subjects (diabetic mellitus type 2) with HbA_1C_.

	Length (in cms)		Transverse dimension (in cms)		DLM (in cms)		DDM (in cms)	
	R	L	R	L	R	L	R	L
Hb1Ac	0.02	0.21	0.09	0.15	0.19	0.37	0.19	0.19

0–0.2, low correlation; 0.2–0.4, moderate correlation; 04–0.7, high correlation; >0.7, strong correlation.

**Table 4:** Comparison of ultrasonographic measurements of parotid glands in study subjects (type 2 diabetes mellitus) with insulin use (oral hypoglycemic drugs and insulin) and those only with oral hypoglycemic drugs.

Insulin	Length (in cms)				Transverse dimension (in cms)				DLM (in cms)				DDM (in cms)			
	R		L		R		L		R		L		R		L	
	No	Yes	No	Yes	No	Yes	No	Yes	No	Yes	No	Yes	No	Yes	No	Yes
N	45	5	45	5	45	5	45	5	45	5	45	5	45	5	45	5
Mean	4.77	4.42	4.69	4.73	3.18	3.52	3.27	3.84	1.41	1.36	1.52	1.2	1.29	1.85	1.27	1.81
S.D.	0.84	0.42	0.90	0.92	1.18	1.07	1.26	1.83	0.57	0.33	1.22	0.31	0.56	0.34	0.54	0.37
*p* value	0.49		0.7		0.51		0.33		0.14		0.05^*∗*^		0.06		0.01^*∗*^	

^*∗*^Significant *p* value.

## Data Availability

The data supporting the results of this study were obtained from the Department of Oral Medicine and Radiology, Government Dental College and Research Institute, Bangalore, Karnataka, India. The data used are included within the article and are also available upon request to the corresponding author.
